# A propensity matched comparison of return to work and quality of life after stenting or coronary artery bypass surgery

**DOI:** 10.1136/openhrt-2015-000322

**Published:** 2016-01-13

**Authors:** Annette M Maznyczka, James P Howard, Amerjeet S Banning, Anthony H Gershlick

**Affiliations:** 1 Kings College London, London, UK; 2 Department of Medical Sciences, University College London, London, UK; 3 International Centre for Circulatory Health, National Heart and Lung Institute, London, UK; 4 Department of Cardiovascular Sciences, University of Leicester and NIHR Leicester Cardiovascular Biomedical Research Unit, University Hospitals of Leicester NHS Trust, Glenfield Hospital, Leicester, UK

## Abstract

**Objectives:**

We sought to determine (1) return to work (RTW) rates, (2) long-term employment (>12 months postprocedure), (3) time taken to RTW, and (4) quality of life (QoL), in patients treated with percutaneous coronary intervention (PCI) or coronary artery bypass grafting (CABG).

**Methods:**

Questionnaires regarding RTW were sent to 689 PCI and 169 CABG patients who underwent PCI or CABG at University Hospitals of Leicester Trust, UK, from May 2012 to May 2013. QoL was also measured using the European QoL 5-dimensions questionnaire (EQ-5D). Responses from patients employed preprocedure were analysed using multivariate logistic regression. Propensity score-matching was further used to compare similar patient populations receiving PCI or CABG.

**Results:**

The response rate was 38% (235 PCI and 88 CABG patients). 241 respondents (75%) were employed preprocedure. Of these 162 (93%) PCI and 51 (77%) CABG patients returned to work, whereas 147 (85%) PCI and 41 (62%) CABG patients were still employed at >12 months postprocedure. After propensity analysis, there was no significant difference between PCI and CABG patients in RTW, long-term employment, nor QoL. The median time taken to RTW was 6 weeks after PCI and 13 weeks after CABG (p=0.001). The effect remained significant after multivariate analysis (p=0.001) and propensity analysis (p=0.001).

**Conclusions:**

In this first propensity score-matched study comparing RTW and QoL after PCI or CABG strict propensity matching indicates that RTW or QoL, is similar for PCI or CABG, albeit the number of matched pairs was small. There are differences, however, in delay in RTW.

**Key questions**

**What is already known about this subject?**

- Previous studies have reported earlier return to work (RTW), but generally similar long-term employment, after percutaneous coronary intervention (PCI) compared with coronary artery bypass grafting (CABG). While some studies have reported no difference in quality of life (QoL) after PCI or CABG, others have reported better long-term QoL after CABG. However, previous studies did not use propensity matching to strengthen causal inferences, and most were prior to the current era of drug eluting stents and short post-PCI in-patient stay.

**What does this study add?**

- This is the first propensity score-matched study comparing RTW and QoL after either PCI or CABG surgery in the contemporary era. Contemporary PCI when compared to CABG surgery predicts earlier RTW. However, after propensity analysis there was no difference between contemporary PCI or CABG surgery in RTW, long-term employment or QoL. The propensity matching which was strict may have limited the comparisons. The results suggest younger age, not having diabetes mellitus, good ejection fraction and not being self-employed are the strongest predictors of RTW.

**How might this impact on clinical practice?**

- RTW and QoL concerns should not drive decision-making regarding selection of PCI or CABG surgery for patients requiring revascularisation. However, from a patient perspective knowing that time taken to RTW is longer after CABG surgery than after PCI may be an important issue to be aware of.

## Introduction

Coronary heart disease can cause premature disability, resulting in socioeconomic issues.^1^ A better understanding of return to work (RTW) and quality of life (QoL) after percutaneous coronary intervention (PCI) and coronary artery bypass grafting (CABG) may help inform patients better, and could provide metrics for patients and physicians to understand longer term social outcomes.

Widely variable RTW rates have been reported in previous studies, ranging from 17% to 90% after CABG and 56% to 98% after PCI with angioplasty, using bare metal stents (BMS) or first generation drug eluting stents (DES).^2^ Factors reported to influence RTW and/or QoL after PCI or CABG surgery include: low socioeconomic status,^3^ unmarried status,^4^ job dis-satisfaction,^5^ pretreatment employment status,^6^ age,^4^ ^7^ ^8^ preprocedural angina^9^ and left ventricular function.^7^ It has been suggested that the strongest predictors of RTW after PCI are: age, sociopsychological and occupational factors.^4^

The limitations of previous studies were their being set in the previous balloon angioplasty or BMS era and are of historical value only. Furthermore, while some beliefs may be intuitive, previous observational studies of RTW did not use propensity score-matching to minimise selection bias and strengthen causal inferences.

The purpose of this study was to test for any differences in RTW and QoL between contemporary PCI and CABG, in a propensity score-matched population. Specific objectives were to describe: (1) RTW rates, (2) employment at >12 months postprocedure, (3) time taken to RTW, (4) QoL and (5) determine which factors influence RTW and QoL, after either PCI or CABG.

## Methods

### Participants

Patients who underwent PCI or CABG surgery, at University Hospitals of Leicester Trust, were identified from a prospectively collected database. To allow for at least 1 year of follow-up, the time period chosen was 1 May 2012 to 1 May 2013. Of the 2323 patients identified, 1465 patients were excluded for the following reasons: age >65 years (n=1196, 51.5%), death (n=65, 2.8%), concomitant valve surgery (n=164, 7.1%), unsuccessful PCI (n=22, 0.9%), or non-UK, or prison address (n=18, 0.8%). Thus 858 patients were deemed eligible for inclusion in the study (criteria: age ≤65 years (on the basis of probability of not being age-retired), CABG surgery, or PCI procedure for acute or stable indication). Questionnaires were sent by postal mail, in August 2014, to 169 patients who had undergone CABG surgery and 689 patients treated with PCI. Data return was supplemented with that from the hospital database, to determine left ventricular ejection fraction (EF) and body mass index results for each participant. Power calculations, derived from the results of previously published studies, were deemed unlikely to be meaningful, due to the wide range of RTW rates in previous publications.

### RTW assessments

**Figure 1:**
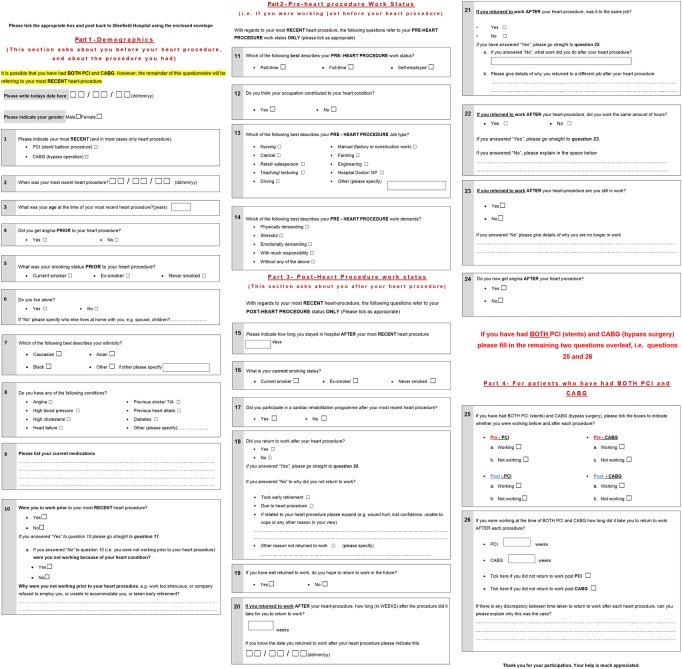
Return to work (RTW) questionnaire.

RTW patterns were assessed using a questionnaire designed in three sections (figure 1). The first focused on demographic factors and questions related to the procedure; the second section on preprocedure work status and the third section focused on postprocedure work status. Questionnaires were analysed anonymous to procedure to minimise bias, by allocating a number to each questionnaire, corresponding to the patient's hospital number and then entering these into a separate database. Long-term employment was defined as ‘still employed >12 months postprocedure’.^10^

### QoL assessments

QoL was assessed using the standardised European QoL 5-dimensions questionnaire (EQ-5D).^11^ The 5 item EQ-5D QoL questionnaire was used, rather than the 19 item Seattle Angina Questionnaire, or the 36 item SF-36 questionnaire, to reduce respondent time burden and since EQ-5D questionnaire results can be easily quantified to detect treatment-related differences.

The EQ-5D questionnaire comprised two sections. The first part contained five questions reflecting five health dimensions: mobility, self-care, usual activity, pain or discomfort and anxiety or depression. Participants were asked to respond to these questions by marking the most appropriate of three possible response levels (1: no problems; 2: some problems; or 3: extreme problems). The second part was the visual analogue scale, that is, a vertical scale that ranged from 0 (worst imaginable health status) to 100 (best imaginable health status). Participants were asked to assess their health state by drawing a line to the appropriate point on the scale.

### Statistical analysis

Baseline characteristics and reported summary statistics are presented as number and percentages for categorical data, and for continuous data as mean±SD, or median with range. Time to RTW is presented as median with range in weeks. Statistical comparison between groups was performed using χ^2^ test, or Fisher's exact tests where appropriate for categorical data and t test for continuous data. The Mann-Whitney test was performed for comparison of non-parametric data. p value <0.05 was considered to represent statistical significance.

Responses from patients employed preprocedure were analysed using multivariate logistic regression. To allow for potential confounding factors between treatments that could influence RTW and QoL, propensity score matching was performed. The following factors were included in the propensity score model; age, gender, procedural urgency, EF <30% and preprocedural job characteristics (self-employed, physical job, stressful job, emotionally demanding job and job with much responsibility). Owing to the significant differences in baseline characteristics between the PCI and CABG groups, calliper matching was used to ensure that each matched variable was within ¼ SD of the matched partner's. The purpose of the propensity score-matching strategy was to reduce confounding effects of these variables, and strengthen causal inferences. Statistics were performed using R V.3.1.2, and propensity score analysis was performed using the ‘MatchIt’^12^ and ‘Zelig’^13^ packages. Patients with missing values were excluded from the univariate, multivariate and propensity analyses.

## Results

### Participants

**Figure 2:**
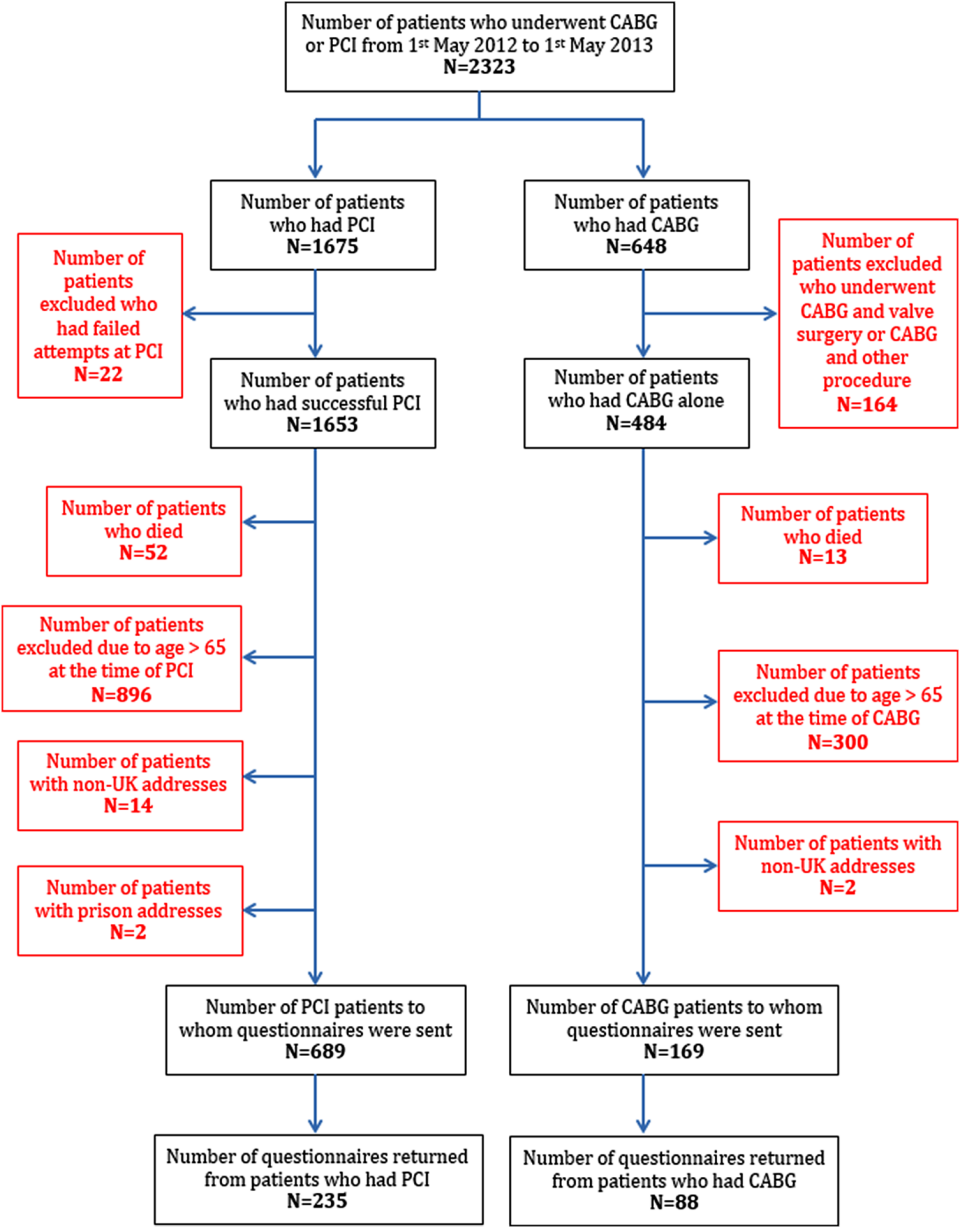
Flow of participants through the study.

Out of 858 participants eligible for inclusion in the study, questionnaires were returned by 323 participants (235 PCI patients and 88 CABG patients), a response rate of 38%. Regardless of this response rate the results are generalisable, since there were no formal power calculations and selection bias of returns was unlikely. The flow of participants through the study is illustrated in figure 2. The overall median time interval from undergoing revascularisation to completing the questionnaire was 21 months (range: 12–27 months).

### Descriptive data

The demographics and procedural characteristics for respondents who were employed preprocedure (n=241) and either did RTW (n=213) or did not RTW (n=28) postprocedure are shown in table 1. The percentages in table 1 represent the proportion of respondents employed preprocedure who did not RTW (column 2), or did RTW (column 3), with that particular characteristic. The demographics and procedural characteristics for respondents who were employed preprocedure and either employed, or unemployed, >12 months postprocedure are shown in table 2. The percentages in table 2 represent the proportion of respondents employed preprocedure who were either unemployed >12 months postprocedure (column 2), or still employed >12 months postprocedure (column 3), with that particular characteristic.

**Table 1:** Demographics for respondents who were employed preprocedure, and either did or did not return to work postprocedure

Characteristic	Employed preprocedure and did not return to work (N=28)	Employed pre procedure and returned to work (N=213)	p Value from univariate analysis
Male, N (%)	21 (75)	192 (90)	0.024
Age at procedure (mean±SD)	61.5 (51–65)	57 (34–65)	0.0003
CABG, N (%)	15 (54)	51 (24)	0.002
PCI, N (%)	13 (46)	162 (76)	0.002
Acute procedure, N (%)	19 (68)	129 (61)	0.457
DM, N (%)	13 (46)	39 (18)	0.001
BMI (mean±SD)	29±4	28±5 (5 missing)	0.141
MI, N (%)	20 (71)	115 (54)	0.086
Smoking post procedure, N (%)	1 (4) (1 missing)	21 (10)	0.317
EF <30%, N (%)	3 (11) (1 missing)	5 (3) (37 missing)	0.005
Lives alone, N (%)	5 (18)	22 (10) (2 missing)	0.249
Self-employed, N (%)	4 (14)	44 (21) (3 missing)	0.00004
Preprocedure job physically demanding, N (%)	13 (46)	80 (38) (3 missing)	0.397
Preprocedure job stressful, N (%)	15 (54)	112 (53) (3 missing)	0.981
Preprocedure job emotionally demanding, N (%)	4 (14)	45 (21) (3 missing)	0.384
Preprocedure job with much responsibility, N (%)	9 (32)	87 (41) (2 missing)	0.359
Cardiac rehabilitation (N, %)	17 (61)	136 (64) (2 missing)	0.699

Percentages represent proportion of respondents who did not return to work (column 2) or returned to work (column 3) post-PCI/CABG with that particular characteristic.

BMI, body mass index; CABG, coronary artery bypass grafting; DM, diabetes mellitus; EF, ejection fraction; MI, myocardial infarction; PCI, percutaneous coronary intervention.

**Table 2:** Demographics for respondents who were employed preprocedure, and were either still working >12 months postprocedure, or unemployed >12 months postprocedure

Characteristic	Employed preprocedure and unemployed >12 months postprocedure (N=51)	Employed preprocedure and still working >12 months postprocedure (N=188)	p Value from univariate analysis
Male, N (%)	42 (82)	169 (90)	0.143
Age at procedure (median+range)	62 (42–65)	56 (34–65)	0.0000001
CABG, N (%)	25 (49)	41 (22)	0.0002
PCI, N (%)	26 (51)	147 (78)	0.0002
Acute procedure, N (%)	33 (65)	113 (60)	0.550
DM, N (%)	18 (35)	34 (18)	0.010
BMI (mean±SD)	29±5	28±5 (5 missing)	0.085
MI, N (%)	28 (55)	105 (56)	0.904
Smoking postprocedure, N (%)	4 (8) (1 missing)	18 (10)	0.733
EF <30%, N (%)	3 (7) (5 missing)	5 (3) (33 missing)	0.026
Lives alone, N (%)	9 (18)	17 (9) (2 missing)	0.091
Self-employed, N (%)	8 (16)	39 (21) (3 missing)	0.018
Preprocedure job physically demanding, N (%)	22 (43)	70 (38) (3 missing)	0.493
Preprocedure job stressful, N (%)	30 (59)	96 (52) (3 missing)	0.380
Preprocedure job emotionally demanding, N (%)	9 (18)	40 (22) (3 missing)	0.536
Preprocedure job with much responsibility, N (%)	15 (29)	81 (43) (2 missing)	0.071
Cardiac rehabilitation, N (%)	30 (59)	120 (65) (2 missing)	0.815

Percentages represent proportion of respondents who are unemployed (column 2) or remain employed (column 3) >12 months post-PCI or CABG with that particular characteristic.

BMI, body mass index; CABG, coronary artery bypass graft; DM, diabetes mellitus; EF, ejection fraction; MI, myocardial infarction; PCI, percutaneous coronary intervention.

Of respondents employed preprocedure data were missing for the following variables: living alone (n=2), body mass index (n=5), smoking status postprocedure (n=1), EF (n=38), preprocedure job type (n=3), participation in cardiac rehabilitation (n=2) and preprocedure job physically demanding (n=3).

Of respondents employed preprocedure data were missing for the following outcomes: still employed >12 months postprocedure (n=2) and time taken to RTW (n=32). Furthermore, 37 respondents did not answer any questions in the QoL questionnaire, despite filling in the RTW questionnaire, thus data were missing from these 37 respondents on the QoL outcomes.

### Outcome data

#### Return to work

**Figure 3:**
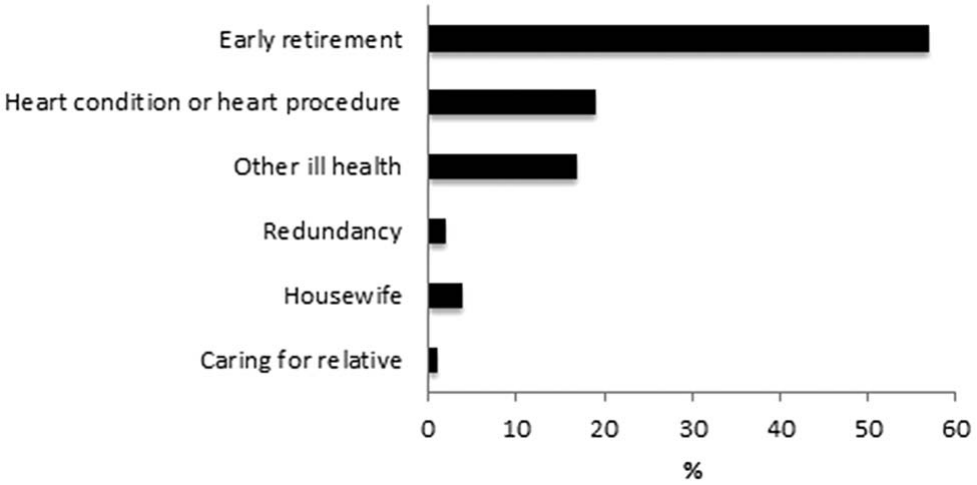
Reasons for unemployment post procedure. CABG, coronary artery bypass grafting; PCI, percutaneous coronary intervention.

Overall 82 of all 323 respondents (25%) were unemployed preprocedure. Ninety six of 323 respondents (30%) were unemployed postprocedure. Only 14% (n=13) of respondents unemployed postprocedure wanted to RTW in the future. Early retirement was the most frequent reason for unemployment postprocedure (57%) (figure 3). Other self-reported reasons for unemployment postprocedure were: (1) the ‘underlying heart condition’, or the revascularisation procedure (19%), (2) other ill-health (17%), (3) redundancy (2%), (4) being a housewife (4%) and (5) caring for a relative (1%).

Two hundred and forty-one respondents (75%) were employed preprocedure. Of these 88% returned to work. Overall 31% (n=74) of respondents who were employed preprocedure thought their job contributed to their heart condition.

Factors that were significantly associated with RTW after multivariate analysis (MVA) of the unmatched cohort were younger age (p=0.001), not having diabetes mellitus (DM) (p=0.011), EF >30% (p=0.035) and not being self-employed (p=0.001).

Out of respondents who underwent PCI and were employed preprocedure 162 (93%) returned to work and 13 (7%) did not (p=0.002). Out of respondents who underwent CABG and were employed preprocedure, 51 (77%) returned to work and 15 (23%) did not (p=0.002), suggesting patients are more likely to RTW after PCI compared to CABG. Out of the respondents who were employed preprocedure and returned to work, 51 (24%) underwent CABG and 162 (76%) underwent PCI. However, type of revascularisation procedure was not associated with RTW after MVA (p=0.402).

After using caliper-matched (¼ SD) propensity scores to select a balanced cohort of 32 pairs, there was no significant difference between PCI and CABG patients in RTW (p=1.000) (table 3).

**Table 3:** Table comparing PCI and CABG, with respect to return to work rates, employment >12 months postprocedure and time taken to return to work

	CABG	PCI	p Values from multivariate analysis	p Values from propensity analysis
Employed preprocedure and returned to work, N (%)	51 (77)	162 (93)	0.402	1.000
Employed preprocedure and still working >12 months postprocedure, N (%)	41 (62)	147 (85)	0.165	0.756
Median time taken to return to work (weeks) (range)	13 (2–52)	6 (0.14–78)	0.001	0.001

CABG, coronary artery bypass grafting; PCI, percutaneous coronary intervention.

#### Long-term employment

Overall 21% (n=51) of respondents, who were employed preprocedure, were not working >12 months postprocedure. After MVA younger age (p=0.000001) and not having DM (p=0.018) remained significantly associated with long-term employment.

Of respondents who underwent PCI and were employed preprocedure 147 (85%) were still employed >12 months postprocedure, (p=0.0002). Out of respondents who underwent CABG and were employed preprocedure 41 (62%) were still employed >12 months postprocedure, whereas 25 (38%) were not (p=0.0002). Out of the respondents who were still employed >12 months postprocedure, 147 (78%) were treated with PCI and 41 (22%) underwent CABG. However, after MVA of the unmatched cohort there was no significant difference between PCI and CABG in long-term employment (p=0.165).

Likewise, after using propensity scores to select a balanced cohort of 32 pairs, there was no significant difference between PCI and CABG in long-term employment (p=0.756) (table 3).

#### Time taken to RTW

The median time taken to RTW was 6 weeks (range: 0.14–78) after PCI and 13 weeks (range: 2–52) after CABG (p=0.001). The effect remained significant on MVA (p=0.001) and after propensity analysis (p=0.001) (table 3).

#### Quality of life

There were no significant differences in self-reported health state scores between PCI and CABG procedures at median follow-up (table 4).

**Table 4:** Self-reported EQ-5D QoL scores for respondents treated with either CABG surgery or PCI, and who were employed preprocedure

Quality of life domain	CABG patients employed preprocedure (n=66)	PCI patients employed preprocedure (n=175)	p Values from multivariate analysis	p Values from propensity analysis
Mobility problem, N (%)	12 (18) (5 missing)	30 (17) (32 missing)	0.772	0.755
Self-care problem, N (%)	0 (5 missing)	6 (3) (32 missing)	0.261	0.321
Usual activity problem, N (%)	18 (27) (5 missing)	37 (21) (32 missing)	0.756	0.761
Pain/discomfort problem, N (%)	25 (38) (5 missing)	50 (29) (32 missing)	0.343	0.794
Anxiety/depression problem, N (%)	13 (20) (5 missing)	53 (30) (32 missing)	0.159	1.000
Health state score (median+range)	80 (20–100) (5 missing)	80 (24–100) (32 missing)	0.267	0.558

Percentages represent proportion of respondents undergoing CABG (column 2) or PCI (column 3) with that specific quality of life domain.

CABG, coronary artery bypass grafting; EQ-5D QoL European QoL 5-dimensions questionnaire quality of life; PCI, percutaneous coronary intervention.

MVA of the unmatched cohort revealed that factors significantly associated with worse self-reported health state scores were living alone (p=0.041), DM (p=0.003), and having a physical job (p=0.005). After MVA of the unmatched cohort: Caucasians had fewer pain/discomfort problems (p=0.015); diabetics had more problems with mobility (p=0.047) and self-care (p=0.021); living alone or acute procedure was associated with anxiety/depression problems (p=0.035 and p=0.015, respectively); and those self-employed or with physical jobs had more usual activity problems (p=0.006 and p=0.0002, respectively).

Propensity analysis of a balanced cohort of 32 pairs likewise revealed no significant differences between PCI and CABG for self-reported health state scores (p=0.558) and for the five EQ-5D QoL domains: problems with pain/discomfort (p=0.794), anxiety/depression (p=1.000), self-care (p=0.321), usual activity (p=0.761) and mobility (p=0.755).

## Discussion

### Main findings

Restoration of normal social-economic functional capacity, particularly RTW, is important but under-researched. The impact of revascularisation procedure on RTW and QoL, was the purpose of this study since all things being equal, socioeconomic factors could influence decision-making around choice of procedure.

Using data from a single high volume interventional and surgical centre, we documented RTW, long-term employment, time taken to RTW and QoL of patients undergoing either PCI or CABG procedures using DES, in the contemporary era. As might be expected recovery to enable work return was shorter after PCI, than after CABG, evidenced by earlier RTW, which can be considered socioeconomically beneficial. However, after propensity analysis there was no difference between contemporary PCI or CABG in overall RTW, long-term employment, or QoL, in this subset of patients. The propensity-score matching allows for potential confounding factors that may influence RTW and QoL postprocedure to be balanced between the two non-randomised cohorts. However, factors that may have had a bearing on the choice of revascularisation strategy (such as patient choice, complexity of coronary disease/ high SYNTAX score leading to CABG, or comorbidities that may result in higher surgical risk leading to complex PCI) cannot be accounted for using propensity matching. Hence, while this process would increase the robustness of any comparison between the PCI and CABG cohort, all of the confounding factors may not be fully accounted for between groups. Some of these factors may also impact on recovery postprocedure, or ability to RTW (eg, a residual ischaemic burden postrevascularisation may impact on ability to regain a group 2 licence). In this particular study, due to the differences in baseline factors, there was a reduction in the number of respondents following propensity matching, which in itself may limit detection of statistically significant differences in QoL or RTW parameters. However, if the propensity analysis holds then RTW and QoL should not drive decision-making regarding selection of a revascularisation procedure.

### Comparison with other studies

Similar to previous studies,^7^ ^14^ we have confirmed that in the contemporary era patients RTW sooner after PCI than after CABG, but long-term employment is similar after the two procedures. Unlike some previous studies,^15^ which reported better long-term QoL after CABG than after PCI, we demonstrated no difference in QoL between the two procedures, in the contemporary era. Other studies have also reported no difference in QoL between PCI and CABG.^16–19^ Our findings are strengthened by the fact that we used propensity matching to minimise confounding.

Detectable variables associated with RTW after MVA were younger age, not having DM, EF >30% and not being self-employed. Similarly, variables associated with long-term employment after MVA of the unmatched cohort, were younger age and not having DM. The former is to be expected, but the latter is interesting and requires some consideration. Longer-term complications of suboptimally controlled DM, such as retinopathy, neuropathy, renal impairment or peripheral vascular disease may be responsible for discontinued employment in the longer-term. However, this needs confirmation in prospective longitudinal studies of employment in patients with DM. Variables associated with worse self-reported health state scores after MVA of the unmatched cohort were living alone, DM, and having a physical job. The predictors of RTW and QoL outcomes that we observed were consistent with previous studies. In addition we have shown that DM may be an important determinate of functional status in this group.

The findings of this study may be considered intuitive. Timing of RTW after a medical procedure, is governed by many factors, including the intrinsic rate of physical recovery, for example, healing of a sternal incision, physician advice, employer policies, social factors and job characteristics. It is expected that the less invasive nature of PCI would enable more rapid convalescence and thus allows a patient to resume normal activities sooner than after CABG surgery. Nonetheless, from a patient perspective knowing that the median time taken to RTW after revascularisation may be at least twice as long after CABG surgery than after PCI (13 weeks vs 6 weeks, p=0.001) may be important enough for the patient to be made aware of this prior to having a procedure.

### Limitations

The findings need to be interpreted in light of the study limitations. First, the propensity matched cohort was small. However, the reason for this is that caliper-matched propensity scores were used, ensuring patients were matched within one quarter of a SD for each criterion. This was necessary due to the highly disparate baseline characteristics of the PCI and CABG groups; the traditional ‘nearest neighbour’ method for propensity matching resulted in a larger but poorly-matched cohort from which it was difficult to draw conclusions. However, obtaining RTW data can be difficult even from clinical trial data, as excluding those patients who are likely retired (>65 years of age) reduces the number of eligible participants. This in turn can affect the ability to detect differences between groups for some of the outcome measures, which may account for the disparity seen in longer-term QoL scores between our PCI/CABG propensity matched cohort and those reported in previous studies where propensity matching was not used.^15^

It is feasible that factor selection for the propensity analysis may mask differences and underestimate the less invasive nature of PCI. However, even when different combinations of variables were used to match the cohort for the propensity analysis, no differences in the main conclusions of this study were detected.

A potential limitation is that a large number of patients were excluded from the 2323 patients who had revascularisation procedures at the single centre between May 2012 and May 2013. This was necessary to address the aims of the study. Patients >65 years old were excluded due to possible work ineligibility and potential age-related functional status impairments. Patients who were unemployed preprocedure were logically excluded from the univariate analysis, MVA and propensity analysis, due to the fact that the main aim of the present study was to analyse RTW patterns.

The difference in proportions of missing QoL outcome data between PCI and CABG groups (table 4) may also potentially introduce bias, for example, the sickest patients may not have completed the QoL questionnaires. However, in a retrospective study such as this, questionnaires are often incompletely filled in by participants, thus this limitation cannot be circumvented fully.

### Conclusion

This is the first propensity-matched study comparing RTW and QoL after PCI or CABG procedures. The results suggest younger age, not having DM, EF >30% and not being self-employed are the strongest predictors of RTW. Contemporary PCI when compared to CABG, after propensity matching, appears to have no impact on RTW or QoL, in this subset of patients, although factor selection for the propensity analysis may mask differences and underestimate the less invasive nature of PCI. Irrespective, PCI patients RTW earlier than CABG patients.

## References

[1] Leal J, Luengo-Fernandez R, Gray A Economic burden of cardiovascular diseases in the enlarged European Union. Eur Heart J 2006;27:1610–19. 10.1093/eurheartj/ehi733 16495286

[2] McGee HM, Graham T, Crowe B Return to work following coronary artery bypass surgery or percutaneous transluminal coronary angioplasty. Eur Heart J 1993;14:623–8. 10.1093/eurheartj/14.5.623 8508856

[3] Denvir MA, Lee AJ, Rysdale J Influence of socioeconomic status on clinical outcomes and quality of life after percutaneous coronary intervention. J Epidemiol Community Health 2006;60:1085–8. 10.1136/jech.2005.044255 17108307 PMC2465496

[4] Isaaz K, Coudrot M, Sabry MH Return to work after acute ST-segment elevation myocardial infarction in the modern era of reperfusion by direct percutaneous coronary intervention. Arch Cardiovasc Dis 2010;103:310–16. 10.1016/j.acvd.2010.04.007 20619241

[5] Fiabane E, Argentero P, Calsamiglia G Does job satisfaction predict early return to work after coronary angioplasty or cardiac surgery? Int Arch Environ Health 2013;86:561–9. 10.1007/s00420-012-0787-z 22684974

[6] Niles NW II, Vandersalm TS, Cutler BS Return to work after coronary artery bypass operation. J Thorac Cardiovasc Surg 1980;79:916–21. 6768935

[7] Hlatky MA, Boothroyd D, Horine S Employment after coronary angioplasty or coronary bypass surgery in patients employed at the time of revascularisation. Ann Intern Med 1998;129:543–7. 10.7326/0003-4819-129-7-199810010-00006 9758574

[8] Panasewicz A, Pedersen SS, Veenhuis JG Health-related quality of life in the elderly three years after percutaneous coronary intervention. EuroIntervention 2013;9:373–81. 10.4244/EIJV9I3A60 23872651

[9] Spertus JA, Salisbury AC, Jones PG Predictors of quality-of-life benefit after percutaneous coronary intervention. Circulation 2004;110:3789–94. 10.1161/01.CIR.0000150392.70749.C7 15596563

[10] Matty S Predicting likelihood of long-term unemployment: the development of a UK jobseekers’ classification instrument. Working paper No 116 Department for Work and Pensions, 2013.

[11] EuroQoL Group. EuroQol—a new facility for the measurement of health-related quality of life. Health Policy 1990;16:199–208. 10.1016/0168-8510(90)90421-9 10109801

[12] Package ‘MatchIt’ 2015 (accessed: 18 May 2015). http://cran.rproject.org/web/packages/MatchIt/MatchIt.pdf

[13] Package ‘Zelig’ 2015 (accessed: 18 May 2015). http://cran.r-project.org/web/packages/Zelig/Zelig.pdf

[14] Allen JK, Fitzgerald ST, Swank RT Functional status after coronary artery bypass grafting and percutaneous transluminal coronary angioplasty. Am J Cardiol 1990;66:921–5. 10.1016/0002-9149(90)90926-R 2220613

[15] Borkon AM, Muehlebach GF, House J A comparison of the recovery of health status after percutaneous coronary intervention and coronary artery bypass. Ann Thorac Surg 2002;74:1526–30; discussion 1530 10.1016/S0003-4975(02)04063-8 12440603

[16] Hlatky MA, Boothroyd DB, Melsop KA Medical Costs and quality of life 10 to 12 years after randomisation to angioplasty or bypass surgery for multivessel coronary artery disease. Circulation 2004;110:1960–6. 10.1161/01.CIR.0000143379.26342.5C 15451795

[17] Abdallah MS, Wang K, Magnuson EA, et al., FREEDOM Trial Investigators. Quality of life after PCI vs CABG among patients with diabetes and multivessel coronary artery disease: a randomized clinical trial. JAMA 2013;310:1581–90. 10.1001/jama.2013.279208 24129463 PMC4370776

[18] Rumsfeld JS, Magid DJ, Plomondon ME, et al., Department of Veterans Affairs Angina With Extremely Serious Operative Mortality (AWESOME) Investigators. Health-related quality of life after percutaneous coronary intervention versus coronary bypass surgery in high-risk patients with medically refractory ischemia. J Am Coll Cardiol 2003;41:1732–8. 10.1016/S0735-1097(03)00330-9 12767656

[19] Wahrborg P Quality of life after coronary angioplasty or bypass surgery 1-year follow-up in the Coronary Angioplasty versus Bypass Revascularization Investigation (CABRI) trial. Eur Heart J 1999;20:653–8. 10.1053/euhj.1998.1237 10208785

